# Improving the hospital waste management at the Farabi hospital in Malekan -Iran: An action research study

**DOI:** 10.1016/j.heliyon.2023.e17695

**Published:** 2023-07-03

**Authors:** Saber Azami-Aghdash, Mahdi Sayadzadeh, Ali Ashtari, Naser Derakhshani, Zahra Sedaei, Ramin Rezapour

**Affiliations:** a Tabriz Health Services Management Research Center, Tabriz University of Medical Sciences, Tabriz, Iran; b Health and Environment Research Center, Tabriz University of Medical Sciences, Tabriz, Iran; c Health Management and Economics Research Center, Health Management Research Institute, Iran University of Medical Sciences, Tehran, Iran; d Student Research Committee, Tabriz University of Medical Sciences, Tabriz, Iran

**Keywords:** Improvement, Intervention, Hospital waste management, Action research

## Abstract

Hospital waste poses numerous concerns for both human health and the environment. Using an action research technique, this study attempts to improve waste management at the Farabi Hospital in Malekan city-Iran. In 2020, integrated (quantitative-qualitative) action research was done. For action research, the Simmons model was employed. First, a list of significant issues was found during the waste management process evaluation using a standard checklist and brainstorming with hospital officials and workers. The identified issues were prioritized using a prioritization matrix. Then, after consulting with hospital officials, 11 interventions were designed and implemented over six months. Finally, waste management performance was re-evaluated. Average knowledge of the participants about hospital waste management (HWM) standards was improved significantly (64 ± 13.8 before the training, 84.6 ± 20.6). General waste production was reduced by 27.7% in terms of garbage bags and 23.4% in terms of waste weight (95.5 kg–73.1 kg), respectively. Infectious waste output was reduced by 22.8% in the number of garbage bags and 32.1% in the weight of waste (57.5 kg–39 kg). The rate of compliance with HWM criteria was improved from 10 to 33 items. Although the interventions in this study improved the HWM to an acceptable level, more interventions and ongoing monitoring are required. The study's findings also show that an action research strategy might address a wide range of issues and weaknesses in hospitals and related facilities.

## 1 Introduction

Waste generated in the community as an inherent part of human life is one of the most serious issues affecting public health and the environment [1,2]. Medical Waste (MW) is classified as hazardous waste because of its poisonous and pathogenic components, which include pathological, pharmacological, chemical, and radioactive substances [3]. In addition to their responsibility for treating patients, delivering health services, increasing well-being, and improving public health, hospitals generate Medical Waste (MW), which is a severe problem for health and the environment [4]. Based on the risk potential, hospital waste is divided into two categories: The first type is non-hazardous waste, which accounts for approximately 85% of all waste generated [5]. The remaining 15% is classified as a hazardous material because it could be infectious, poisonous, or radioactive [5]. According to research conducted in Ethiopia, MW collectors were exposed to any sharp item. 85.2% of individuals who reported sharp injuries had needle stick injuries [6]. Farzadkia et al. (2013) investigated Hospital Waste Management (HWM) in one of Tehran's hospitals in a study. The daily solid waste generation was roughly 1750 kg. There was 54.13%, 46.43%, and 2.41% of general, infectious, and sharp waste in each active bed, respectively. Inadequate waste separation was to blame for the large volume of infectious waste [7].

The understanding of the waste management status may be influenced by healthcare waste indicators [8]. Various indicators have been explored by researchers to assess and improve healthcare waste management [9,10]. The generation rate of sharp and infectious waste and the health workers knowledge of healthcare waste management standards are the most important indicators used to assess the waste management system [8,11].

In addition to using indicators that have significant achievements and outcomes, the researchers have developed an innovative approach called “action research” [12,13]. Action research is a powerful tool for bringing about organizational and professional transformation. In recent years, action research, or practical research, has been advocated in health systems to analyze and solve current challenges and problems, and it is one of the most important types of study [14,15].

According to available documents and information, the state of WM in Iran's hospitals is poor [16]. Inadequate training of engaged people, poor separation, inappropriate temporary storage of HW, inadequate sterilization and disposal of infectious waste, use of incinerators to disinfect HW, and environmental pollution are all WM issues in the hospital [16,17]. Using an action research study approach, this project attempts to improve the WM of the Farabi Hospital in Malekan City.

## 2 Method

The research is a mixed (quantitative-qualitative) action research study [18] that was conducted in 2020 at Farabi Hospital in Malekan City. To perform action research, simple and realistic Simmons models were employed. This model consists of six major steps, which were carried out in the following order.

### 2.1 Step 1- Discovering the subject or issue

The Farabi Hospital in Malekan City was chosen for upgrading after consulting with responsible officials and the WM research team.

### 2.2 Step 2- data collection

A reliable and valid checklist, which was developed by the Ministry of Health and Medical Education, was used to provide a quantitative and comprehensive evaluation of the WM process.

This checklist is split into two sections. The first section featured questions about the center's general information, and the second section included specific inquiries (44 questions in 4 sections). The checklist was prepared by seeing the WM process, analyzing documentation, and interviewing hospital personnel. The WM process flowchart was then created in the Farabi Hospital for a more accurate evaluation of WM (Appendix 1).

Based on the quantitative evaluation and brainstorming with hospital officials and employees, difficulties, and weaknesses in WM were identified; additionally, the list of problems was prioritized using the prioritization matrix from the perspectives of hospital authorities and staff. The brainstorming section's target population includes personnel working in WM-related hospital wards. The sampling method was comparable to that used in most qualitative research, which used target-based sampling. Samples that provide the greatest information were chosen for this sampling approach.

Inclusion criteria for staff included more than two years of experience in the Farabi hospital, working in one hospital ward connected to HW, and having the desire and consent to participate in the study. In the case of officials, however, having a history of responsibility in HWM in the previous two years, as well as the desire and consent to engage in the study, were inclusion criteria. Eleven officials and workers from various departments participated in the problem-solving brainstorming session. After introducing the process's rules and concepts, participants were given 20 min to focus on the brainstorming approach. The issues raised by meeting attendees were then documented. Using the prioritization matrix, the problems extracted in the previous phase were prioritized. Participants scored the five indicators “Problem severity,” “Problem extent,” “Problem side effects,” “Problem-solving ability,” and “Problem-solving effectiveness” from 1 (lowest) to 5 (highest) to prioritize each problem. The average scores of the participants were then used to compute the scores of each of the indicators.

### 2.3 Step 3- planning

This part of the study was conducted in two stages: designing interventions and prioritizing interventions. First, appropriate interventions were designed to improve the HWM through a focused group discussion based on the hospital conditions and the problems identified and prioritized in the previous stage. At the beginning of the session, with the participant's consent, their viewpoints were noted and recorded to investigate. To better manage the session, the researchers used a semi-structured interview guide form. The information extracted from the focused group discussion session was manually analyzed, summarized, and reported using the content analysis method. Theme analysis is a technique for detecting, analyzing, and reporting patterns (themes) in text that has several uses in qualitative data research [[19], [20], [21], [22]].

The data was analyzed by two academics. As a result, the suggested interventions derived from individuals' explanations and assertions were listed and sent for prioritizing. We used the prioritization matrix to prioritize the interventions after creating them from the standpoint of the study participants. Participants ranked each intervention based on four criteria: acceptance, effectiveness, cost, and time, assigning each a score ranging from 1 (lowest) to 5 (highest). Finally, the study team created a table of operational plans to undertake interventions based on the current conditions, identified problems, and interventions.

### 2.4 Step 4- action/intervention

The preceding stage's plans and interventions were carried out as planned. The most critical interventions are the purchase of a destroyer with a shredder, the provision of essential training to staff and clients, and the purchase of necessary equipment such as binaries, standard bins in adequate quantities, and other goods.

### 2.5 Step 5- evaluation

A quantitative evaluation approach was used to evaluate the effects of interventions and programs implemented in the action research form. The same checklist was used in the second step for quantitative evaluation. Also, changes in the production volume of infectious and general wastes before and after six months of interventions were measured and compared.

Descriptive statistics including mean, frequency, and percentage was used to describe the data. To assess the effect of the intervention on HWM improvement and compare the status of HWM standards adherence after and before intervention implementation, we used inferential statistics. Before performing the statistical test, the normality of data was assessed using the Kolmogorov-Smirnoff test. The paired *t*-test was used as a parametric test and Wilcoxon as none parametric one. Data analysis was performed through SPSS 16 software. The significance level of statistical tests was 0.05.

### 2.6 Step 6- feedback

A report of the action research process was prepared, and intervention plans and results were written in detail and presented to the Farabi Hospital's officials to provide feedback.

## 3 Results

### 3.1 The waste management process's performance evaluation

**Fig. 1:**
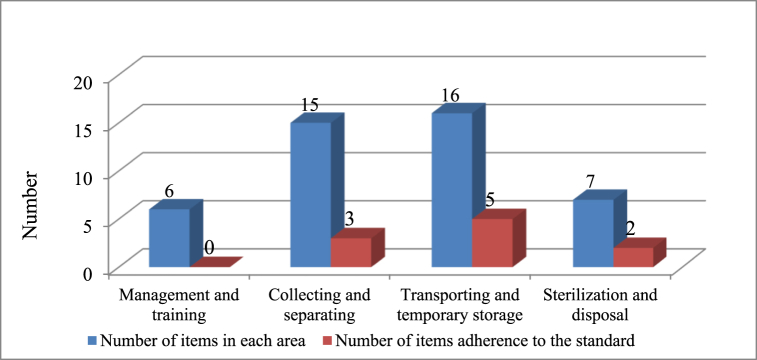
The adherence rate with hospital waste management standards in the Farabi hospital over the four area.

The WM process's performance evaluation results showed that many checklist items were not in adherence to standards in the hospital, and only a limited number of them (10 out of 44 items) were considered (Fig. 1). In this stage, a flowchart of the WM implementation process in the Farabi hospital was designed to evaluate WM properly (Appendix 1).

Identifying and prioritizing the problems in waste management of the Farabi Hospital in Malekan city based on the results of quantitative evaluation in the previous stage and the brainstorming's outcome, finally, 20 severe problems and challenges in the Farabi hospital concerning WM were identified and entered into the priority matrix. The problem prioritization scores, which are the mean scores of 11 people, are presented in Table 1.

**Table 1:** Prioritization matrix of problems in waste management of Farabi Hospital.

No.	Problems List	Severity of the problem^a^	Extent of the problem^a^	Complications of the problem^a^	Problem solving feasibility^a^	The effectiveness of problem solving^a^	Total points
1	Low staff awareness of segregation and importance of segregation of infectious waste (lack of training)	4.5	4	4	5	4.5	22
2	Lack of waste management program in the hospital	3	3.5	3.5	4	3.5	17.5
3	Lack of manuals or training packages to teach environmental health and safety and occupational health.	4	3.5	4	4.5	4	20.5
4	Lack of healthy, lined, stainless, washable waste bins with appropriate volume and number	4	3.5	4.5	4	4.5	20.5
5	Lack of observance of color in segregation and non-principled segregation of production waste in sections	4.5	4	4.5	5	4	22
6	Failure of service personnel to use personal protective equipment in the collection of production waste	4	4.5	4.5	4	4.5	21.5
7	Failure to collect garbage bags on time in terms of filling volume (3.4 bags volume)	3.5	4	4.5	4.5	3.5	20
8	Transportation of infectious waste with normal waste manually and leakage of wine in the environment and pollution	4.5	4	5	5	4.5	23
9	Lack of proper labeling of waste bags	4.5	4	4.5	4	4	21
10	Lack of space for carrying enough waste in the wards	4.5	4	4.5	5	4.5	22.5
11	Do not use between to transport waste	4.5	4	4.5	4.5	4	21.5
12	Failure to observe the color of the gaps in the storage of waste separately in the temporary waste chamber	4	3.5	4	4	3.5	19
13	Failure to maintain general cleanliness of the waste disposal site and disposal site	3.5	4	4	4.5	4	20
14	Undesirable temporary waste storage room and disposal site	4	3.	3.5	3	3.5	17.5
15	Failure to comply with the standard for the duration of waste storage in temporary storage	3.5	3	4	4	3.5	18
16	Lack of disinfection and cleaning between wastes for waste storage	3.5	4	3.5	4	3.5	18.5
17	Lack of disposal device and use of incinerator in the hospital	4	4.5	5	3.5	5	22
18	Lack of monitoring and self-declaration documents of hazardous waste	3	3.5	3	4	3.5	17
19	Inadequate disposal of non-hazardous waste (incinerated)	4	3.5	4.5	4	4	20
20	Lack of experienced and full-time disposal user	4	4.5	4.5	4	4.5	21.5

a Score from 1 to 5.

Design, prioritize, and implement interventions to improve waste management performance in the Farabi Hospital in Malekan.

The results of intervention design and prioritization are presented in Table 2.

**Table 2:** Prioritization matrix of interventions to improve the performance of waste management at Farabi Hospital.

No.	Interventions List	Acceptance^a^	Effectiveness^a^	Cost^a^	Time^a^	Point Total
1	Holding a training workshop for employees	4.5	4	4.5	5	18
2	Prepare waste management manuals for employees	4.5	4	4.5	4	17
3	Preparing pamphlets for clients	4.5	4	4	4.5	17
4	Pay incentives for employees who meet most standards.	4	4.5	3	3.5	15
5	Prepare a report/letter about the use of incinerators to the relevant authorities	4	4.5	5	5	18.5
6	Prepare standard waste bins according to the needs of different wards of the hospital	4	4.5	4	4	16.5
7	Preparation of colored labels to observe the segregation of waste	4	4.5	4.5	4.5	17.5
8	Prepare a trolley to transport the waste to the temporary chamber	4	4	4	4.5	16.5
9	Creating competition between departments to meet more standards	3.5	4	3.5	3	14
10	Designate a safe and experienced user for the location of the disposal site	3.5	4.5	4	4	16
11	Provision and supply of appropriate personal protective equipment for the waste staff	4	4.5	4	4	16.5
12	Procurement and supply of standard disposal device	4.5	4.5	3.5	4	16.5
13	Increase service personnel to manage the waste sector	3.5	4.5	3.5	3.5	15
14	Improving the location of the disposal site	4.5	4	3.5	3.5	15.5
15	Transfer to the private sector	3.5	4	4	3.5	15

a Score from 1 to 5.

At this stage, the researchers and hospital officials suggested 15 interventions. Eventually, 11 interventions were selected to improve HWM based on the hospital officials' and researchers' average scores and views. An action plan was developed to implement each intervention. The most important interventions are as follows.

1 A one-day workshop, “Waste Management in Hospital,” was held in coordination with the University Research and Development Coordination Centre (RDCC).
2 The Inadequacy of waste disposal conditions in the Farabi hospital due to the lack of waste disposal devices was reported to various officials, including the Vice-Chancellor for Treatment deputy of Tabriz University of Medical Sciences.
3 A WM training handbook was compiled for hospital staff.
4 Educational pamphlets on HWM were prepared for clients.
5 Standard waste bins were prepared according to the hospital's needs in different wards.
6 Colored labels were prepared to separate waste.
7 Bins were prepared to transport waste to a temporary room based on the hospital's needs.
8 Experienced users were designated for the eradication site.
9 Proper personal protective equipment was provided to the department's service staff and safe users.
10 Standard waste disposal devices were prepared and installed.
11 The waste incineration building was discharged, a standard demolition site map from the university's treatment deputy was provided, and the demolition site was improved (Appendix 2).

## 4 Reassessment results

Satisfaction results from the workshop and pre-and post-test on knowledge of waste management.

A “Hospital Waste Management Workshop” was arranged, and 71 clinical staff members from Malekan's Farabi Hospital attended. The audience was taught about different types of HW, the HWM process, the criteria, and methods of executive management of HW and related wastes, waste separation standards, waste color coding, waste label titles, and the principles and methods of waste transportation, storage, and disposal. Finally, 60 of the 71 workshop participants completed the satisfaction forms. The form has 19 questions divided into four sections. According to the satisfaction survey results, approximately 86% of the participants were happy with the course. Members of the research team created a 10-item, four-choice questionnaire based on the workshop content to assess participants' knowledge before and after the workshops in two stages (immediately after the workshop and three months later). The results showed that the average knowledge of the participants before the start of the workshop was 64 ± 13.8 which increased to 84.6 ± 20.6 and 80.1 ± 15.6 just after the workshop and three months later, respectively (P < 0.05).

## 5 Results related to waste volume reduction

Because most interventions began on September 1, the cut-off point for the outcomes of produced rubbish bags and waste weight in the Farabi hospital was October 1 (the average of the first six months was compared to the average of the second six months). Before the intervention, the average number of garbage bags was 45.1 bags, and the average weight was 95.5 kg per month.

**Fig. 2:**
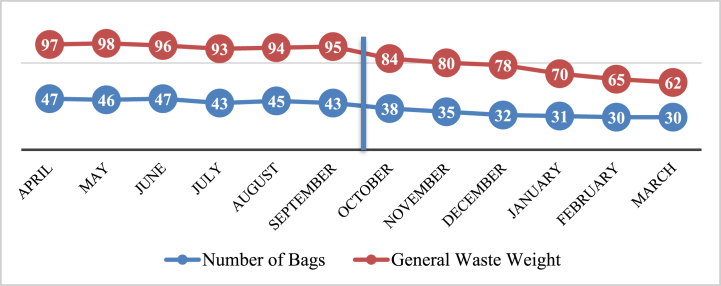
Number of produced garbage bags and weight of produced general waste in the Farabi hospital in 2020.

After the interventions (the second 6 months of the year), the average number of general waste bags decreased to 32.6 cases, and the average weight of general waste decreased to 73.1 kg per month (Fig. 2). This reduction in garbage bags and the weight of general waste were significant in both cases (P < 0.05).

**Fig. 3:**
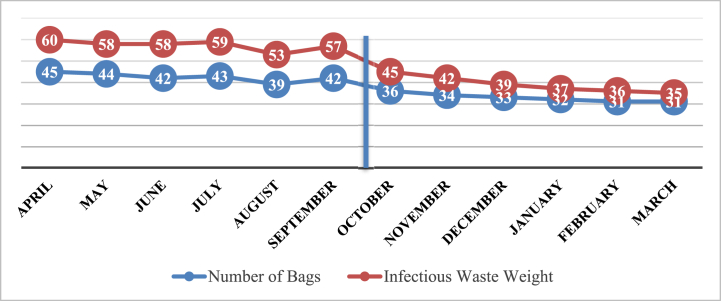
Number of produced garbage bags and weight of produced infectious waste in the Farabi hospital in 2019.

Before the intervention, the average number of contaminated bags was 42.5 bags, and the average weight was 57.5 kg per month in infectious garbage. Following the interventions (during the second half of the year), the average number of contaminated waste bags was reduced to 32.8, and the average weight of infectious garbage was reduced to 39 kg per month (Fig. 3). There was a considerable reduction in the number of garbage bags and the weight of infectious waste (P < 0.05).

**Fig. 4:**
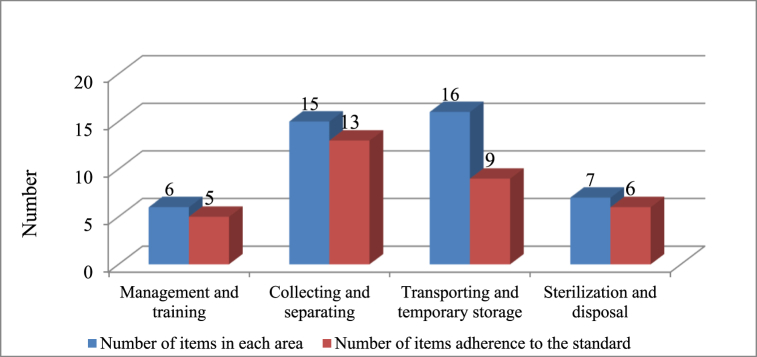
The adherence rate with HWM standards in The Farabi hospital over the four area after the intervention.

According to the quantitative evaluation results (using an evaluation checklist), after the treatments, the adherence rate to HWM norms increased from 10 to 33 items. The highest level of standards adherence was in Collecting and separating (^13^/_15_ = 86%) and Sterilization and disposal (^6^/_7_ = 85%) area (Fig. 4).

**Fig. 5:**
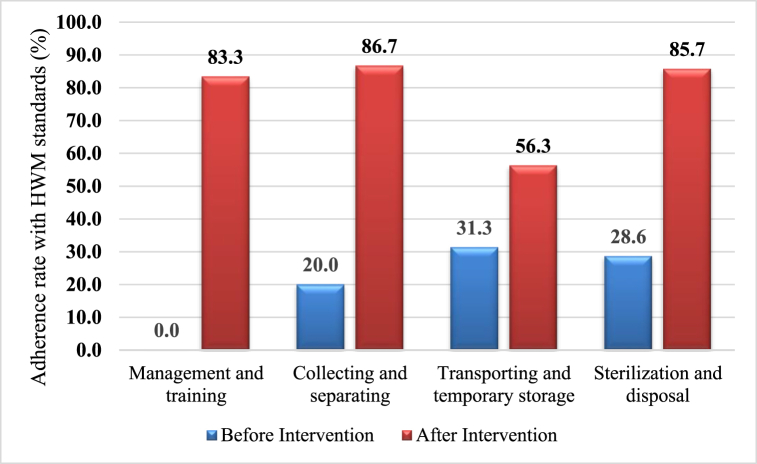
Comparison of the adherence rate with HWM standards before and after the implementation of waste management improvement interventions.

Fig. 5 shows the percentage of WM items' implementation in the Farabi hospital before and after WM promotion interventions. As mentioned, there has been a significant increase in all areas, especially in management, training, and disposal.

### 5.1 Other results of the study

Secondary outcomes were acquired based on observations and opinions of beneficiaries that were not measured and were not involved in the core goals of the study. The main goals are mentioned below.

- Improvement in the importance and sensitivity to HWM's issues among hospital managers and staff
- Reduction in air pollution due to the replacement of the disposal device with the waste incinerator.
- Reduction in the hospital and city environment's pollution caused by HW's ashes using incinerators. After the transportation from the hospital environment. There were problems with the disposal site.
- Reduction in residents' dissatisfaction who are living near the hospital and reducing risks.

Incinerating garbage smoke inconveniences residents of Mehr residential complexes near the hospital. By solving this problem, the level of risk and dissatisfaction among residents has also decreased.

- Hospital administrators and management admitted that they had been dealing with WM issues for a long time. However, they were unable to attain successful outcomes, whereas the mentioned WM issues were immediately resolved by the scientific method utilized in this work.

## 6 Discussion

The action research method was found to be effective in increasing the standard adherence rate of HWM in this study. Farabi Hospital demonstrated a 52% improvement in HWM standards adherence, as observed in this study. This accomplishment was made possible by major variables such as staff involvement in decision making, evidence-based interventions, and leadership commitment. One of the most important treatments in the current study was to raise hospital personnel's and managers' understanding of the HWM standards and their significance. Vosoughi Nairi et al. (2016) investigated hospital personnel's knowledge, attitude, and performance regarding HWM. The findings demonstrated a poor level of knowledge, attitude, and performance, as well as a moderate degree of performance [23].

Taghipour et al. (2012) evaluated the knowledge, attitude, and performance of Tabriz hospital personnel about HWM; the results revealed that the mean score of knowledge and attitude among hospital staff was different [24]. Physicians and specialists received the highest knowledge and attitude evaluations, while service employees and HW collectors had the lowest scores. The researchers concluded that a high degree of knowledge and awareness is insufficient for carrying out the HWM improvement plan, and that personnel performance should be monitored to improve [24]. Moladoust et al. (2016) studied nurses' understanding of HWM in educational and medical centers associated with Isfahan University of Medical Sciences [25]. The results showed that nurses who had received in-service waste management education had higher mean knowledge scores in the separation and collection, storage, sterilization, and disposal stages than nurses who did not engage in these courses. The researchers found that developing and implementing continuous and in-service waste management education programmed encourages nurses to learn continually in order to increase knowledge, attitude, and skills that can be useful in proper waste management [25]. Based on the current study and earlier papers, several strategies for educating hospital administrators and personnel about sanitary and hospital correct WM concepts are required. Several strategies were utilized in this regard, including hosting workshops, showcasing hospital performance, developing and distributing guidebooks, executing numerous treatments, increasing visits, emphasizing WM, and preparing brochures for clients. Hospital waste separation and collection are the most important phases of hospital waste management. An Egyptian investigation found that waste separation was not done in accordance with standardized standards and criteria [26]. Our findings revealed that, before the intervention, only around 20% of standards had been met in the collection and separation area. Medical waste separation must occur at the point of generation in health care facilities [27]. A lack of source separation, a lack of color coding, a lack of record keeping, and staff irresponsibility are some of the key causes mentioned as producing inadequate separation practices in developing country hospitals [28]. Inadequate source separation might lead to needlestick injuries among hospital waste management staff [29]. As a result, hospitals must provide enough separating apparatus. So, in the Farabi hospital, changes such as providing standard color-coded bags and containers and bin labelling were implemented.

From the participants' perspective, waste management in the Farabi hospital was a major problem. This approach creates odors, smoke, and air pollution, which generally bother people. For this reason, in recent years, Iran and many other countries have outlawed incinerators [30]. The most effective method is to use waste disposal equipment, as most hospitals in the country do [31,32].

One of the most important results of improving WM in the Farabi hospital was a significant reduction in waste volume (general and infectious). A study by Chirol et al. (2007) in Japan found that 75%–90% of HW was general waste and non-hazardous [33]. Taghipour and Mosaferi's (2009) study in Tabriz hospitals showed that general waste accounts for 70.11% of the total HW [34]. At the end of this study (second half of 1398), by some interventions, especially training participants and monitoring waste separation, this rate was reduced to 31.8%. Both the volume of hospital waste and the quantity of garbage bags were reduced by 34% and 40%, respectively. Martin et al. (2017) used the Lean Six Sigma method to undertake an intervention to improve WM in the United States. As a result, the volume and number of waste bags decreased by 12 and 6%, respectively [35]. As a result of the current study and prior studies in this field, it appears that by executing interventions similar to action research or any other sort of intervention, it is feasible to greatly reduce the volume of waste produced, particularly infectious waste. As a result of the negative effects of hospital waste and risk, it is advised that hospital managers plan and implement such measures. The action research method was employed in this study to improve waste management at the Farabi hospital. Specific principles and guidelines must be followed for this technique. One of the fundamental principles is to maximize beneficiary participation. Several beneficial and significant individuals were chosen as research partners prior to this study, including the director of the municipal health network, the head of the hospital, and the provincial head of environmental health. In order to entice their opinions and participation in this study, the study's goals and benefits were also communicated to some other significant people who were unable to engage as research partners.

## 7 Conclusion

Hospital waste management standards have considerably improved following the introduction of initiatives in all four areas, particularly management, training, and disposal. The application of the action research technique, as well as employee participation and evidence-based decision-making, may result in the improvement of hospital waste management systems.

Hospital waste management was a neglected subject that required more attention. This study offers a scientific technique for improving hospital waste management at Malekan Hospital that has the potential to be reproduced in other hospitals in Iran or similar nations.

## Author contribution statement

Saber Azami-Aghdash: Wrote the paper; Analyzed and interpreted the data, materials.

Mahdi Sayadzadeh: Wrote the paper, Contributed materials, analysis tools or data.

Ali Ashtari: Wrote the paper, Conceived and designed the intervention.

Naser Derakhshani: Wrote the paper, Analyzed and interpreted the data, materials.

Zahra Sedaei: Wrote the paper, Contributed materials, analysis tools or data.

Ramin Rezapour: Wrote the paper, Conceived and designed the intervention, Analyzed and interpreted the data.

## Funding statement

This study is supported by the 10.13039/501100004366Tabriz University of Medical Sciences.

## Ethics approval

This study was approved in the Research Ethics Committee of Tabriz University of Medical Sciences (ethical code: IR.TBZMED.REC.1398.1169). The methods were performed in accordance with the relevant guidelines and regulations. "Informed consent” was obtained from all study participants.

## Data availability statement

Data will be made available on request.

## Additional information

No additional information is available for this paper.

## Declaration of competing interest

The authors declare that they have no known competing financial interests or personal relationships that could have appeared to influence the work reported in this paper.
