# Supplementary material for: Improving the hospital waste management at the Farabi hospital in Malekan -Iran: An action research study
Source: Heliyon. 2023 Jul 3;9(7):e17695. doi: 10.1016/j.heliyon.2023.e17695 (PMC10359768; doi:10.1016/j.heliyon.2023.e17695)
Supplement: Multimedia component 1 [file mmc1.docx]

Labeling of infectious and general waste bags

Separation of general and infectious wastes

Collection of waste after filling the volume of 3/4 bags

Manual transportation of infectious and general waste

Placing infectious and general waste in temporary storage

Is the collected waste infectious waste?

Putting waste in the incinerator by the disposal operator

Additional File 1: Waste management Follow chart in Farabi Hospital
