# Supplementary material for: Improving the hospital waste management at the Farabi hospital in Malekan -Iran: An action research study
Source: Heliyon. 2023 Jul 3;9(7):e17695. doi: 10.1016/j.heliyon.2023.e17695 (PMC10359768; doi:10.1016/j.heliyon.2023.e17695)

**Additional File 2: Intervention Documents**

Holding a one-day workshop on "Hospital Waste Management" in coordination with the Research Development and Coordination Center (RDCC)
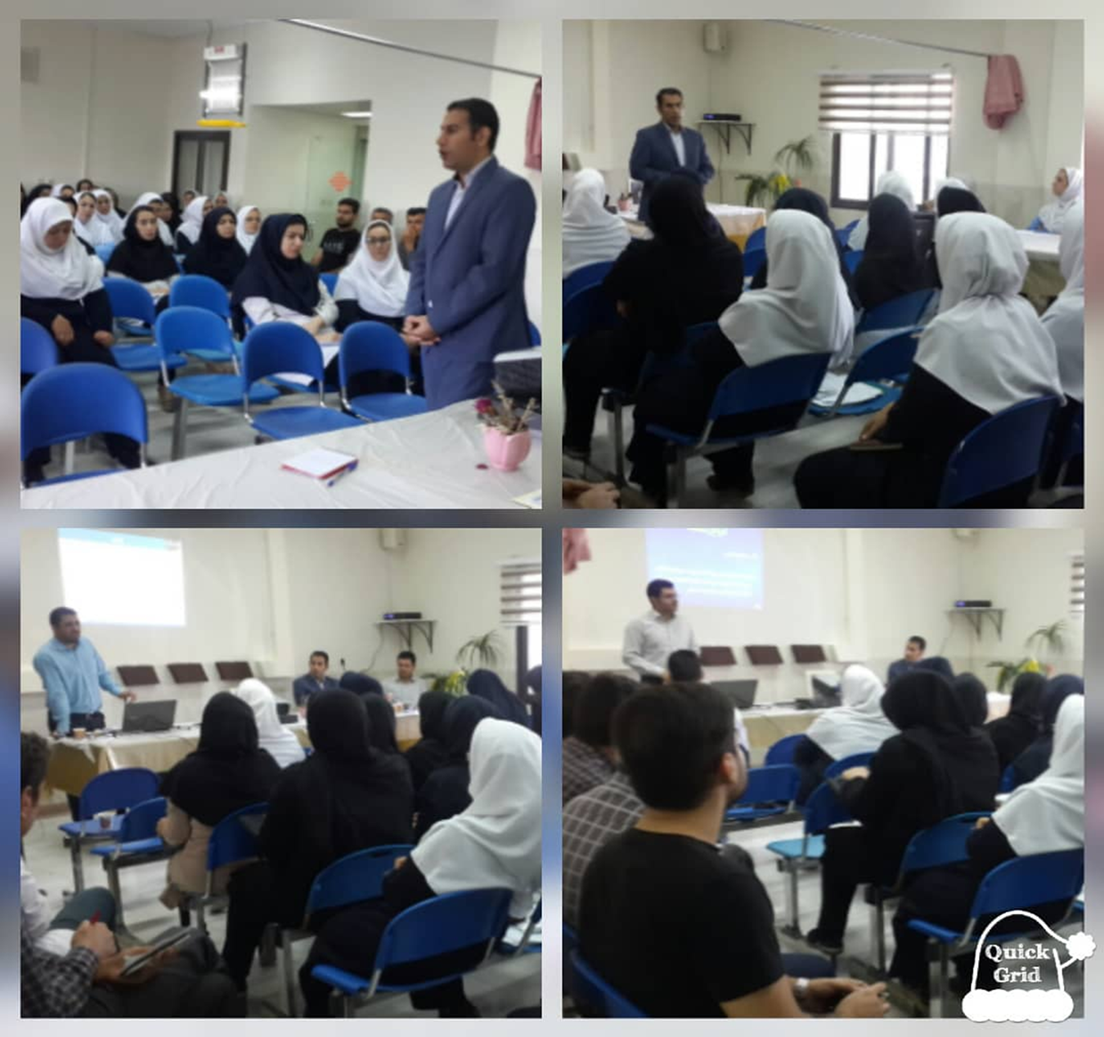


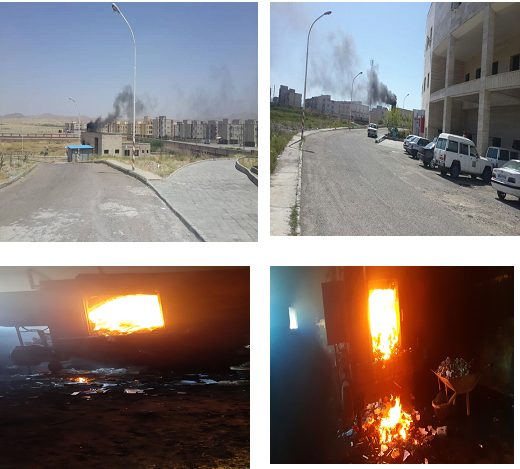


Training manual for waste management


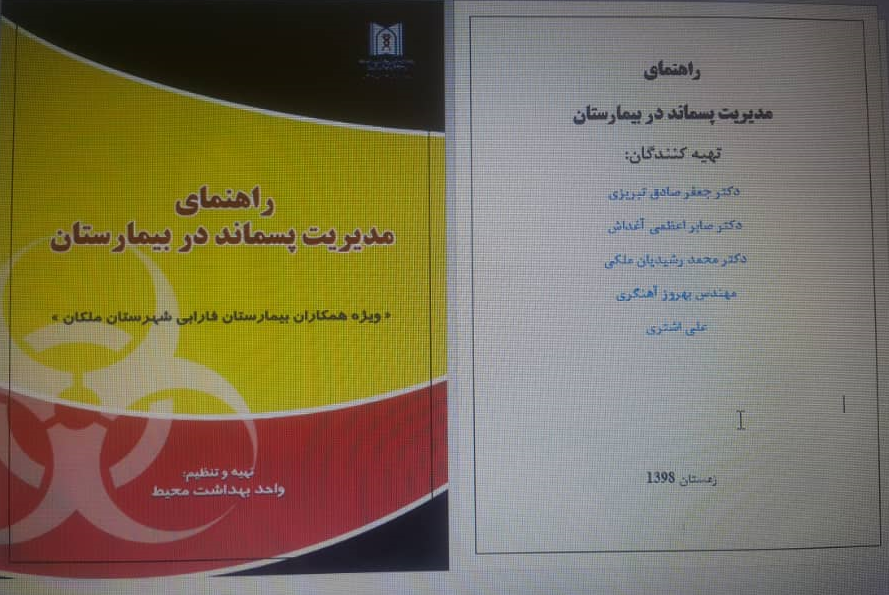


Preparation of educational pamphlet on waste management in the hospital


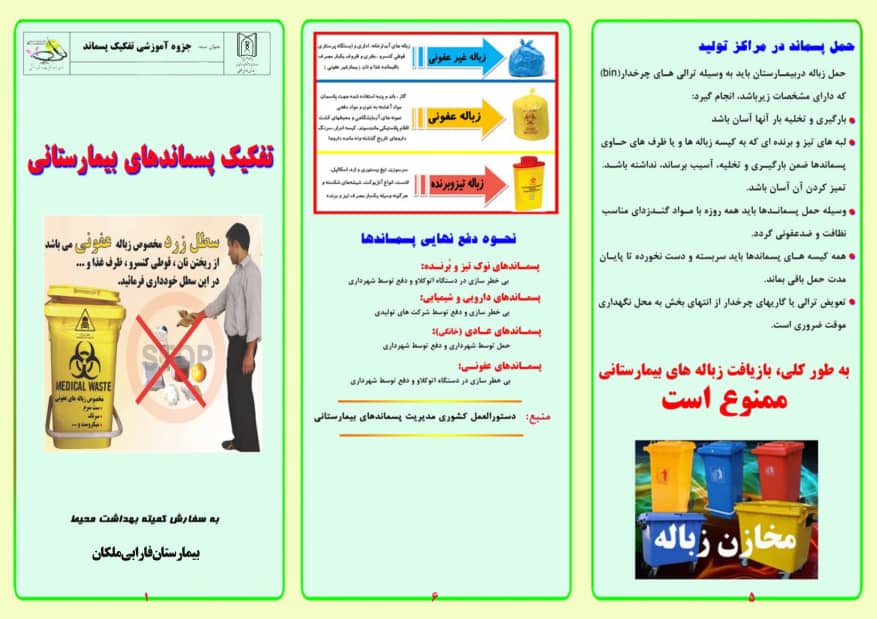


Preparation of standard waste bins according to the needs of different wards of the hospital

| 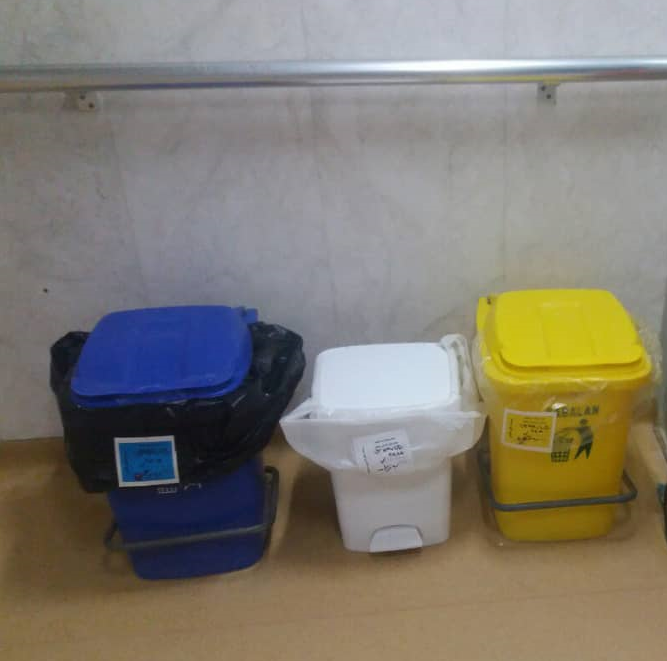 |
| --- |
|  |


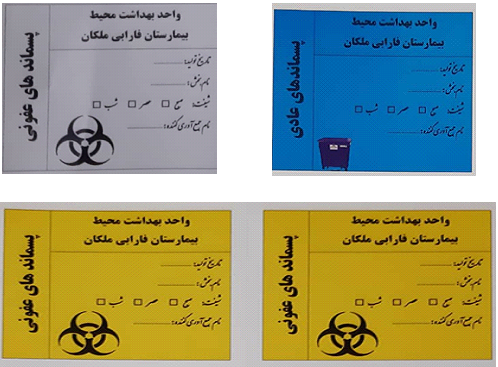
 Preparation of colored labels to observe the segregation of waste

| 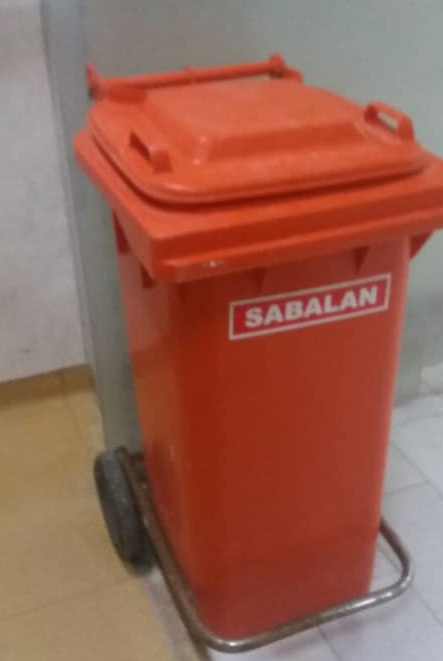 |
| --- |
|  |

Preparing a trolley to transport the waste to a temporary room according to the needs of the hospital

Preparation and installation of standard waste disposal device


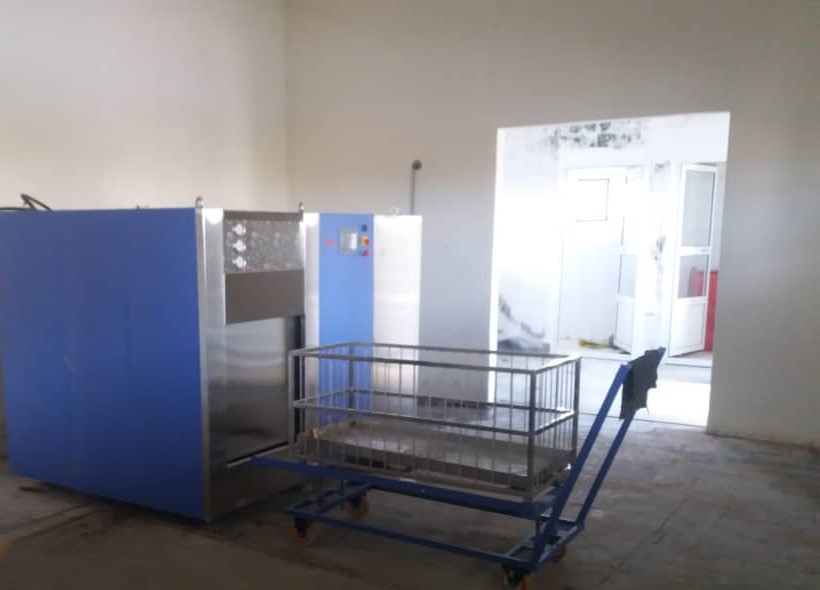


Destruction of waste incineration building, preparation and supply of standard disposal site map and improvement of disposal site


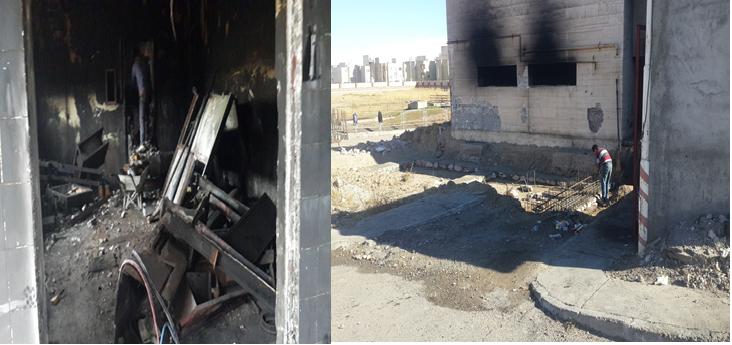

Supplement: Multimedia component 2 [file mmc2.docx]
